# Investigation of the JASMONATE ZIM-DOMAIN Gene Family Reveals the Canonical JA-Signaling Pathway in Pineapple

**DOI:** 10.3390/biology11030445

**Published:** 2022-03-15

**Authors:** Li Ye, Ling Cao, Xuemei Zhao, Xinya Guo, Kangzhuo Ye, Sibo Jiao, Yu Wang, Xiaoxue He, Chunxing Dong, Bin Hu, Fang Deng, Heming Zhao, Ping Zheng, Mohammad Aslam, Yuan Qin, Yan Cheng

**Affiliations:** 1 State Key Laboratory of Ecological Pest Control for Fujian and Taiwan Crops, College of Plant Protection, Fujian Agriculture and Forestry University, Fuzhou 350002, China; yeliyouxiang@139.com (L.Y.); lic792@mail.usask.ca (L.C.); xuemei_310@hotmail.com (X.Z.); gxy019@hotmail.com (X.G.); yekangzhuo@163.com (K.Y.); dengfang@fafu.edu.cn (F.D.); 2 Fujian Provincial Key Laboratory of Haixia Applied Plant Systems Biology, Center for Genomics and Biotechnology, College of Life Science, Fujian Agriculture and Forestry University, Fuzhou 350002, China; jobs.fafu@outlook.com (S.J.); suw376707@gmail.com (Y.W.); hexiaoxue@wh.iov.cn (X.H.); d_chunxing@163.com (C.D.); 1200514014@fafu.edu.cn (B.H.); zhaoheming@whu.edu.cn (H.Z.); zhengping13@mails.ucas.ac.cn (P.Z.); aslampmb1@gmail.com (M.A.); 3 College of Agriculture, Fujian Agriculture and Forestry University, Fuzhou 350002, China; 4 Guangxi Key Lab of Sugarcane Biology, College of Agriculture, Guangxi University, Nanning 530004, China

**Keywords:** JAZ, JA signaling, Phytohormone, pineapple, salt stress

## Abstract

**Simple Summary:**

JASMONATE ZIM-DOMAIN (JAZ) proteins are key components of the jasmonate (JA) signaling pathway in response to biotic and abiotic stresses in plants. Information about the *JAZ* gene family in pineapple (*AcJAZ*) is limited. In this study, 14 *AcJAZ* genes were identified in the pineapple genome. A complete overview of AcJAZ genes is presented, including the chromosome locations, phylogenetic relationships, gene structures, conserved motifs and cis-regulatory elements, and expression patterns at different developmental stages and under various stress conditions, and their possible involvement in diverse functions is suggested. Furthermore, the BiFC analysis revealed direct binary interactions between AcJAZs and crucial JA-signaling regulators in vivo. These results suggest that AcJAZs and other vital players function in the JA-signaling pathway in response to abiotic stresses in pineapple.

**Abstract:**

JASMONATE ZIM-DOMAIN (JAZ) proteins are negative regulators of the jasmonate (JA)-signaling pathway and play pivotal roles in plant resistance to biotic and abiotic stresses. Genome-wide identification of JAZ genes has been performed in many plant species. However, systematic information about pineapple (*Ananas comosus* L. Merr.) *JAZ* genes (*AcJAZs*) is still not available. In this study, we identified 14 *AcJAZ* genes and classified them into five groups along with the Arabidopsis and rice orthologs. The *AcJAZ* genes have 3–10 exons, and the putative AcJAZ proteins have between two and eight conserved regions, including the TIFY motif and Jas domain. The *cis*-acting element analysis revealed that the putative promoter regions of *AcJAZs* contain between three and eight abiotic stress-responsive *cis*-acting elements. The gene-expression analysis suggested that *AcJAZs* were expressed differentially during plant development and subjected to regulation by the cold, heat, salt, and osmotic stresses as well as by phytohormones. Moreover, the BiFC analysis of protein interactions among the central JA-signaling regulators showed that AcJAZ4, AcMYC2, AcNINJA, and AcJAM1 could interact with AcJAZ5 and AcJAZ13 in vivo, indicating a canonical JA-signaling pathway in pineapple. These results increase our understanding of the functions of AcJAZs and the responses of the core players in the JA-signaling pathway to abiotic stresses.

## 1. Introduction

Jasmonates (JAs), the endogenous plant metabolites that exist ubiquitously in plants, primarily include Jasmonic acid (JA), Methyl *cis*-jasmonate (MeJA), (+)-7-iso-jasmonoyl-L-isoleucine (JA-Ile), and its precursors such as OPDA (12-oxo Phytodienoic acid), dinor-OPDA (dinor-12-oxo-Phytodienoic acid), and oxygenated fatty acids (oxylipins). JAs play an important role in regulating plant growth, development, reproduction, and response to biotic and abiotic stresses along with other plant hormones [1,2,3,4]. It has been shown that in tobacco Bright Yellow-2 cells, JAs play a vital role in maintaining cells at the G1 stage by preventing DNA replication [5]. In *Arabidopsis*, wound-induced jasmonates inhibit mitosis and reduce the cell number, leading to stunted plant growth [2]. Consistently, JAs adversely affect leaf growth by repressing cell proliferation and the onset of endoreduplication [6]. JAs also regulate the biosynthesis of ethylene and control the timing of leaf senescence through 1-aminocyclopropane-1-carboxylic acid (ACC) conjugation [7,8]. They are well known as transducers of elicitor signals for the accumulation of plant secondary metabolites [9]. Further, JAs play critical regulatory roles in plant biotic and abiotic stresses, as shown by many studies [1,3,10,11,12,13,14,15,16,17,18].

Previous studies have suggested that JA-Ile is a bioactive jasmonate of the JA-signaling pathway. It has a similar structure and function as coronatine (a bacterial toxin), activated in JA signaling as a substrate [19,20,21]. The basic helix–loop–helix (bHLH) transcription factors, MYC2, MYC3, MYC4, and MYC5, are core transcription factors in the JA-signaling pathway which redundantly promote the expression of JA-responsive genes [22,23,24]. COI1, an F-box protein subunit of the SKP1-CUL1-F-box (SCF) complex with E3 ubiquitin ligase activity, is the receptor of JA-Ile [25,26]. On the other hand, JASMONATE ZIM-DOMAIN (JAZ) proteins are the repressors of JA signaling, inhibiting the expression of JA-responsive genes by repressing the transcriptional activity of MYC2, MYC3, MYC4, and MYC5 [27]. JAZ proteins belong to the TIFY gene family, harboring a TIFY motif (TIF[F/Y]XG), previously known as Zim domain, in the N-terminal region and a Jas or CCT_2 motif (SLX2FX2KRX2RX5PY) in the C-terminal region [28]. In the absence of active JAs, JAZ proteins, through their Jas domains, bind to MYC transcription factors and recruit NINJA (NOVEL INTERACTOR OF JAZ) proteins through their TIFY motif. NINJA proteins further interact with TPL (TOPLESS) proteins, transcription repressors with an EAR domain. The JAZ–NINJA–TPL complex represses the expression of the MYC2/3/4/5-target genes [29]. In the presence of the external or endogenous stimulus, JA-Ile accumulates in plants and is subsequently received by the SCF^COI1^ preceptor [30]. The JA-Ile then mediates the interaction of SCF^COI1^ with JAZ proteins, which are then ubiquitinated by SCF^COI1^. The ubiquitinated JAZ proteins are targeted to 26S proteasomes for degradation, releasing MYC2/3/4/5 and resulting in the expression of JA-responsive genes.

JAZ proteins are the core regulators of the JA-signaling pathway that are also involved in phytohormonal crosstalk [31]. In *Arabidopsis* and rice (*Oryza sativa*), 13 and 15 *JAZ* genes have been identified, respectively. In recent years, genome-wide identification of the *JAZ* gene family has been made in several plants, including *Petunia axillaris* (12), *Petunia inflate* (16), *Triticum aestivum* (14), *Solanum lycopersicum* (13), *Vitis vinifera* (11), *Hevea brasiliensis* (18), and sugarcane (7), and the functions of *JAZ* genes were extensively investigated, especially in *Arabidopsis* [32,33,34,35,36,37]. For example, the decuple *jaz* mutant exhibits enhanced resistance to biotic stresses, attenuated vegetative growth, and reduced the seed setting, suggesting the important functions of *Arabidopsis JAZs* in plant development and stress response [38]. It has also been reported that the *jaz10* knockout mutant has enhanced JA sensitivity and increased susceptibility to DC3000 in *Arabidopsis* [39].

Pineapple (*Ananas comosus* L. Merr.), a perennial monocot belonging to the family *Bromeliaceae*, is one of the most important tropical fruit crops around the world with edible and decorative values [40]. For edible usage, pineapples are rich in vitamins, organic acids, saccharides, and trace elements such as calcium and iron. For decoration, pineapples have a perfect flower-shaped structure, bright and gorgeous color, and long flowering time.

Moreover, pineapple plants have excellent stress tolerance to adverse conditions, including drought and salt stress. Since JA signaling plays a pivotal role in plant response to stresses and JAZ proteins are a core component of JA signaling, it is important to explore the JA-signaling pathway based on the *JAZ* gene family in pineapple (*AcJAZ*). In this study, the genes involved in the JA-signaling pathway were identified in pineapple, and an extensive investigation of the AcJAZs was conducted. These results provide a comprehensive understanding of the JA-signaling pathway in pineapple, which should facilitate further research related to pineapple stress response and tolerance.

## 2. Materials and Methods

### 2.1. Identification of JAZ Gene Family in Pineapple

The protein sequences of pineapple (variety F153) were downloaded from the Pineapple Genomics database (PGD, http://pineapple.angiosperms.org/pineapple/html/index.html (accessed on 20 December 2020)). The proteins harboring TIFY (PF06200) and Jas (PF09425) domains were identified using the HMMER3.0 (http://hmmer.org (accessed on 20 December 2021)) program with default parameters. Furthermore, 13 *Arabidopsis* JAZ (AtJAZ) protein sequences were downloaded from TAIR (http://www.arabidopsis.org/ (accessed on 20 December 2021)), and 15 rice (*Oryza sativa*) JAZ (OsJAZ) proteins were downloaded from TIGR (release 5; http://rice.plantbiology.msu.edu (accessed on Dec. 20, 2021)) [41]. Those sequences were used as queries to search for JAZ proteins against the pineapple protein database using the blastp algorithm with an e-value of 1 × 10^−5^. Then, Pfam (http://pfam.xfam.org/search#tabview=tab1 (accessed on 20 December 2021)), SMART (http://smart.embl.de/smart/batch.pl (accessed on 20 December 2021)) [42], and NCBI CDD (https://www.ncbi.nlm.nih.gov/cdd/ (accessed on 20 December 2021)) were used to determine the correct JAZ proteins in pineapple. The detailed information, including gene location on chromosomes, intron number, and CDS length of *AcJAZ* genes, and the amino acid number, molecular weight, and isoelectric point (pI) of AcJAZ proteins, was acquired from Phytozome v12.1 (http://phytozome.jgj.doe.gov/pz/portal.html (accessed on 20 December 2021)) and ExPasy (http://web.expasy.org/protparam/ (accessed on 20 December 2021)). The information of related chromosome length and *AcJAZ* location was obtained from the PGD, and the location map was constructed by MapChart [43].

### 2.2. Phylogenetic Analysis

The protein sequences of 14 AcJAZs, 13 AtJAZs, and 15 OsJAZs were aligned by MEGA7 using the Clustalw method. Then, the alignment file was used to construct a Neighbor-Joining-based phylogenetic tree with the bootstrap value of 1000. The phylogenetic tree was annotated using the online tool Evolview (https://evolgenius.info//evolview-v2/#login (accessed on 20 December 2021)). All protein sequences used for phylogenetic analysis are listed in Supplementary File S1.

### 2.3. Analysis of Conserved Motif and Gene Structure

To show sequence similarity between JAZ proteins of pineapple, 14 AcJAZ sequences were used to create a phylogenetic tree by MEGA7 and present conserved motifs and gene structures. The MEME software (Multiple EM for Motif Elicitation, http://meme-suite.org/tools/meme (accessed on 20 December 2021)) was used to identify conserved motifs with the maximum searching number set to 15. All motif information of pineapple JAZ proteins is listed in Supplementary File S2. Moreover, the exon–intron organization data were extracted from the Pineapple Genomics database (PGD). The visualization of the phylogenetic tree, conserved motifs, and gene structures was conducted with Tbtools [44].

### 2.4. Analysis of Cis-Acting Elements

The 2000 bp upstream sequences from the translation start codon (ATG) were obtained from the Phytozome for all the AcJAZs and used to analyze the *cis*-acting elements using PlantCARE (http://bioinformatics.psb.ugent.be/webtools/plantcare/html/ (accessed on 20 December 2021)). The following abiotic responsive elements: abscisic acid-responsive element (ABRE, ABRE3a, ABRE4, AT-ABRE), MeJA-responsive elements (CGTCA-motif, TGACG-motif), wound-responsive elements (W-box, WRE3, WUN-motif), ethylene-responsive elements [29], drought-responsive elements (DRE core), salicylic acid-responsive elements (TCA), low-temperature-responsive elements (LTRE), gibberellin-responsive elements (GARE-motif, TATC-box, P-box), auxin-responsive elements (AuRR-core, TGA-element, TGA-box), and defense and stress elements (TC-rich repeats) were searched.

### 2.5. Plant Materials and Stress Treatments

Regenerated plantlets of pineapple (variety MD2) with approximately 10 cm length were transferred to the greenhouse at 25 °C and under 16 h light/8 h dark photoperiod [45]. After 30 days of the transfer, plants/seedlings were treated with cold (4 °C), heat (45 °C), osmotic stress (15% PEG), salt (150 mM NaCl), IAA (100 μM), MeJA (100 μM), ABA (100 μM), SA (100 μM), and 6-BA (100 μM) followed by sample harvesting at 0, 2, 4, 12, and 36 h for RNA extraction [45,46].

### 2.6. Total RNA Extractions and RT-qPCR

E.Z.N.A Total RNA Kit II (OMEGA, Guangzhou, China) was used for total RNA extraction, followed by cDNA synthesis using AMV reverse transcriptase (Takara, Beijing, China). The cDNAs were then used for RT-qPCR analysis using 2 × TranStart Top Green qPCR SuperMix (TransGen Biotech, Beijing, China). A 20 μL reaction which included 8 μL nuclease-free water, 0.5 μL (10 mM) forward primer, 0.5 μL (10 mM) reverse primer, 1 μL cDNA, and 10 μL 2 × TranStart Top Green qPCR SuperMix was used for qPCR in a CFX96 Touch real-time PCR machine with the following parameters: 95 °C for 30 s, 40 repeats of 95 °C for 5 s, and 60 °C for 40 s. The relative expression levels of targets were calculated using the delta–delta Ct method using pineapple housekeeping gene *PP2A* as reference. The online tool IDT (https://sg.idtdna.com/pages/products/qpcr-and-pcr/gene-expression/primetime-primer-only-assays-and-primers (accessed on 20 December 2021)) was used to design real-time PCR primers (Table S1). For expression profiling of the *JAZ* gene family in pineapple, the RNA-seq data for Calyx (C1–C4), Gynoecium (G1–G7), Ovule (O1–O7), Petal (P1–P3), Stamen (S1–S6), Fruit (S1–S7), Flower, Leaf, and Root were used (Accession Number: PRJEB38680) [47].

### 2.7. BiFC Assay

The YFP-N end-tagged pSPYNE and YFP-C end-tagged pSPYCE vectors were used to transform *Agrobacterium tumefaciens* strain GV3101 and then infiltrate tobacco (*Nicotiana benthamiana*) leaves for BiFC (Bimolecular fluorescence complementation) assay. The tobacco infiltration and inflorescence observations were conducted following the procedure reported by Yuan and Xu [48]. Infusion strategies were adopted for vector preparation using CloneExpress II One Step Cloning Kit (Vazyme, Nanjing, China). The primers used in this assay are listed in Table S1.

### 2.8. The Cellular Localization of AcJAZ Proteins

The protein sequences of 14 AcJAZs and 15 OsJAZs were analyzed to predict possible subcellular localization using DeepLoc (https://services.healthtech.dtu.dk/service.php?DeepLoc-1.0 (accessed on 20 December 2021)) and MULocDeep (http://mu-loc.org/ (accessed on 20 December 2021)) [49]. All protein sequences used for the prediction of subcellular localization are listed in Supplementary File S1.

## 3. Results

### 3.1. Fourteen JAZs Were Identified in the Pineapple Genome

**Figure 1:**
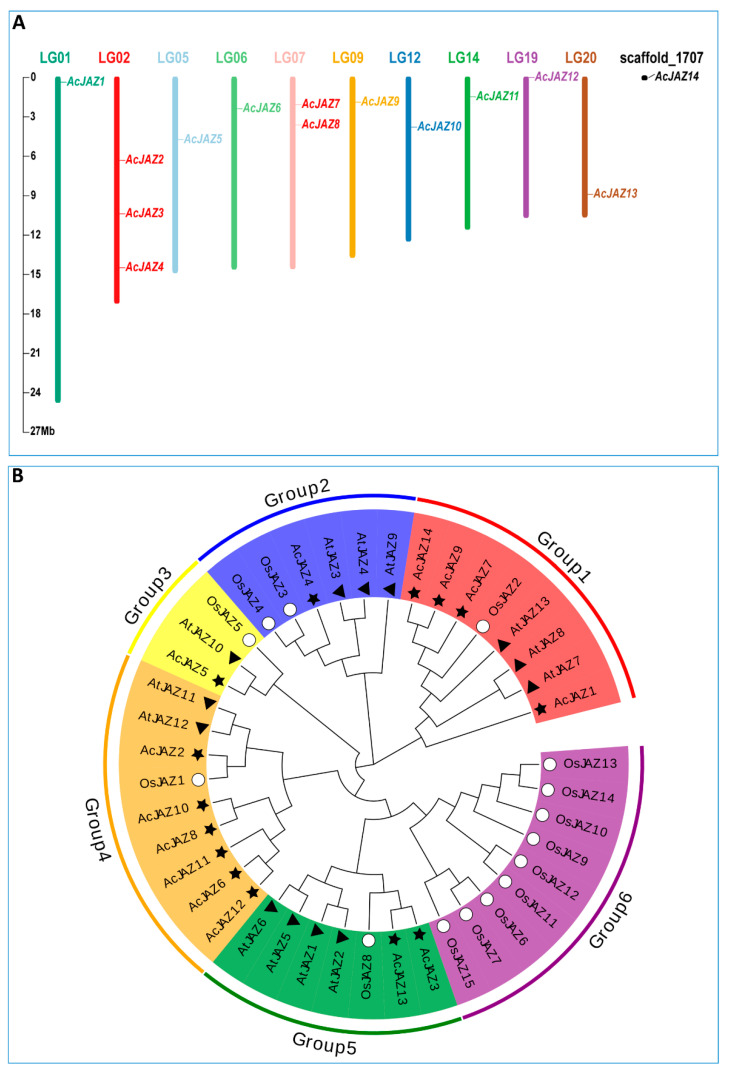
The JAZ gene family in pineapple. (**A**) Chromosomal locations of JAZ genes in pineapple. The respective chromosome number is indicated by different colors at the top of each chromosome. The scale on the left is for chromosomes in megabases (**B**) Phylogenetic relationship of JAZ proteins from *Arabidopsis thaliana*, *Oryza sativa*, and pineapple. The phylogenetic tree was created using the neighbor-joining method with 1000 bootstrap replicates by MEGA 7. The diverse groups of JAZ proteins are marked with different colors. The JAZ proteins of *A. thaliana*, *O. sativa*, and pineapple are represented by black triangles, white circles, and black stars, respectively.

The genome of pineapple variety F153 was used as a reference in this study [50]. In total, 14 JAZ proteins were identified based on BLASTP and HMM searching, and the corresponding genes were designated as AcJAZ1–AcJAZ14 based on their location on pineapple chromosomes from top to bottom, in which AcJAZ1–13 were located on ten pineapple chromosomes (chromosomes 01, 02, 05, 06, 07, 09, 12, 14, 19, 20), and AcJAZ14 was located on an unanchored scaffold_1707 (Figure 1A). Based on the DNA and amino acid sequences, the characteristics of pineapple JAZ genes and the putative proteins, including gene ID, chromosome locations, complete coding sequence (CDS) length, exon numbers, protein length, molecular weight, and isoelectric point (pI) are shown in Table 1. The AcJAZ genes possessed a minimum of three exons, and AcJAZ1 had the maximum (10) exons. The CDSs of AcJAZs ranged from 486 to 1608 bp in length, with the corresponding protein length ranging from 161 to 535 aa, the molecular weight from 16,867.08 Da to 56,897.76 Da, and the isoelectric point between 5.32 and 9.8 (Table 1). 

### 3.2. Protein Sequence Analysis Showed the Phylogenetic Relationship of AcJAZs

The evolutionary relationship among the pineapple JAZ proteins was studied by constructing a phylogenetic tree along with 13 Arabidopsis and 15 rice JAZ protein sequences using MEGA X, following the neighbor-joining (NJ) method (Figure 1B). The JAZ proteins were divided into six groups according to the topology of the phylogenetic tree, and the *AcJAZs* were represented in five groups. In the first group, one *OsJAZ* (*OsJAZ2*), three *AtJAZs* (*AtJAZ7*, *AtJAZ8*, and *AtJAZ13*), and four *AcJAZs* (*AcJAZ1*, *AcJAZ7*, *AcJAZ9*, and *AcJAZ14*) were grouped. *OsJAZ3*, *OsJAZ4*, *AtJAZ3*, *AtJAZ4*, *AtJAZ9*, and *AcJAZ4* belonged to the second group. The third group contained only three genes, i.e., *OsJAZ5*, *AtJAZ10*, and *AcJAZ5*. *OsJAZ1*, *AtJAZ11–AtJAZ12*, and six *AcJAZs (AcJAZ2*, *AcJAZ6*, *AcJAZ8*, *AcJAZ10*, *AcJAZ11*, and *AcJAZ12*) belonged to the fourth group. The fifth group was composed of *AcJAZs* (*AcJAZ3* and *AcJAZ13*), *AtJAZs* (*AtJAZ1*, *AtJAZ2*, *AtJAZ5*, and *AtJAZ6*), and *OsJAZ8*. The sixth group only had *OsJAZs* (*OsJAZ6*, *OsJAZ7*, *OsJAZ9*–*OsJAZ15*) (Figure 1B). The phylogenetic relationship of AcJAZs along with AtJAZs and OsJAZs indicated that those JAZ proteins shared a similarity of protein sequences and a common ancestor.

### 3.3. AcJAZs Are Conserved in Gene Structure and Motif Organization

**Figure 2:**
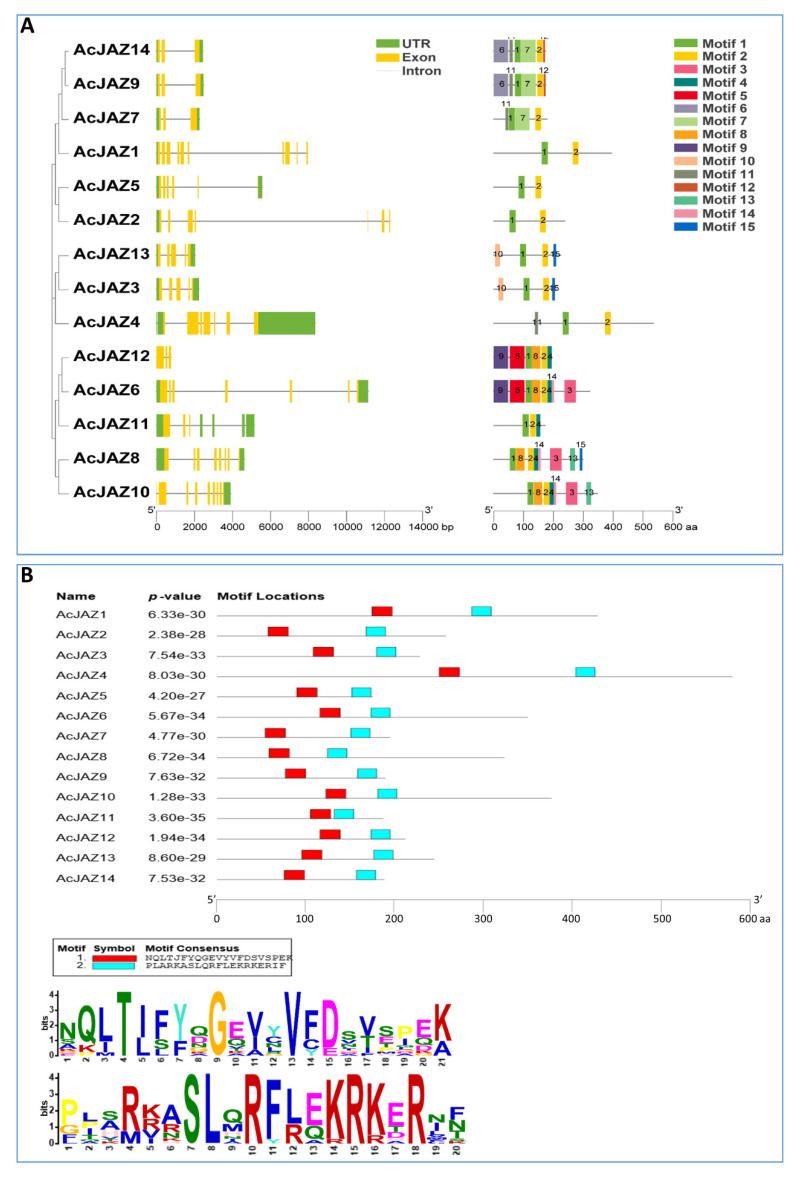
The JAZ proteins in pineapple. (**A**) Phylogenetic relationships, gene structures, and motifs of *JAZ* genes in pineapple. The first lane shows the phylogenetic tree of AcJAZ proteins generated using the neighbor-joining method with 1000 bootstrap replicates by MEGA 7. The second lane shows the exon–intron organization of *AcJAZs*. 5′ and 3′ UTRs, exons, and introns are indicated by green boxes, yellow boxes, and black lines, respectively. The scale at the bottom represents the number of nucleotides. The third lane shows the conserved motifs of Pineapple JAZ proteins detected by MEME software. Different motifs for AcJAZ proteins are indicated by different colored boxes and numbered 1–15. The scale at the bottom represents the number of amino acids. (**B**) Conserved motif 1 and motif 2 in AcJAZ proteins. The upper diagram shows the positions of conserved motif 1 and motif 2 in AcJAZs, and the scale below represents the number of amino acids. The lower diagram shows the consensus amino acids for motif 1 (the upper panel) and motif 2 (the lower panel). The height of an amino acid character indicates proportionality to the sequence conservation.

To understand the gene structure and protein motif in a phylogenetic view, the JAZ proteins of pineapple were used to construct a phylogenetic tree, gene structure, and motif analysis in the same order as in the phylogenetic tree. In the absence of Arabidopsis and rice, the phylogenetic tree showed a similar relationship with the result described above (Figure 2A). According to the pineapple genome release (V3.0), the introns in *AcJAZs* ranged from two to nine, and exons ranged from 3 to 10. *AcJAZ2* had the most extended genomic DNA sequence (12,295 bp) with the longest intron (fourth intron, 9049 bp), while *AcJAZ4* had the shortest nucleotide sequence with the shortest intron (third intron, 53 bp). Most of the *AcJAZ* genes have the 3′ UTR and 5′ UTR annotated, with the exceptions of *AcJAZ10* without 5′ UTR, *AcJAZ1* and *AcJAZ2* without 3′ UTR, and *AcJAZ12* lacking both UTRs. *AcJAZ7*, *AcJAZ9*, and *AcJAZ14* had similar structure and sequence lengths with two introns and three exons.

Protein motifs might be assigned putative functions ofproteins. The motifs of pineapple JAZ proteins were identified using Multiple-Expectation maximization for Motif Elicitation (MEME) software. All AcJAZs harbored between two and eight motifs, while AcJAZ1, 2, 4, and 5 had two motifs (Figure 2A). Motif 1 and motif 2 were present in all AcJAZs, whereas motif 3 and motif 4 were present in five AcJAZs (Figure 2A). Two protein pairs, AcJAZ9/AcJAZ14 and AcJAZ3/AcJAZ13, had the same motif features (Figure 2A and Figure S2). Further, AcJAZ protein sequences were subjected to Pfam analysis to identify the conserved motifs, and we found nine Pfam motifs (tify, Jas_motif, CCT, F-box_4, FXR_C1, GATA, zf-Dof, Fz, and TFIIE-A_C) (Figure S3). Motif 1 and motif 2 of the MEME analysis represented the tify and Jas domains, respectively (Figure 2B), and motif 3 represented the GATA zinc finger domain, which uses four cysteine residues to coordinate a zinc ion. However, no Pfam information was detected for motifs 4 (PKKIRYTVRKEVALR) and 7 (VQAVLLLLGG), and they could be new motifs with unknown functions.

To investigate whether those JAZ proteins are localized in the nucleus, we analyzed the possible subcellular localization of AcJAZs and OsJAZ proteins using DeepLoc (https://services.healthtech.dtu.dk/service.php?DeepLoc-1.0 (accessed on 20 December 2021)) and MULocDeep (http://mu-loc.org/ (accessed on 20 December 2021)). Both methods were consistent with each other in predicting that all 14 AcJAZs were localized in the nucleus (Table S2). As a comparison, we also analyzed 15 rice JAZ proteins. The DeepLoc analysis predicted that all 15 OsJAZs were localized in the nucleus, while MULocDeep predicted that 12 OsJAZs were localized in the nucleus and three OsJAZ were mainly localized elsewhere (Table S3). The prediction score of the nucleus localization of AcJAZ1 and AcJAZ3 is relatively low, while the those of the nucleus localization of AcJAZ2, AcJAZ7, AcJAZ8, AcJAZ9, AcJAZ10, AcJAZ11, AcJAZ12, and AcJAZ14 are rather high, indicating the strong nucleus localization of JAZ proteins of pineapple. 

### 3.4. AcJAZs Are Differentially Expressed in Pineapple Organs

**Figure 3:**
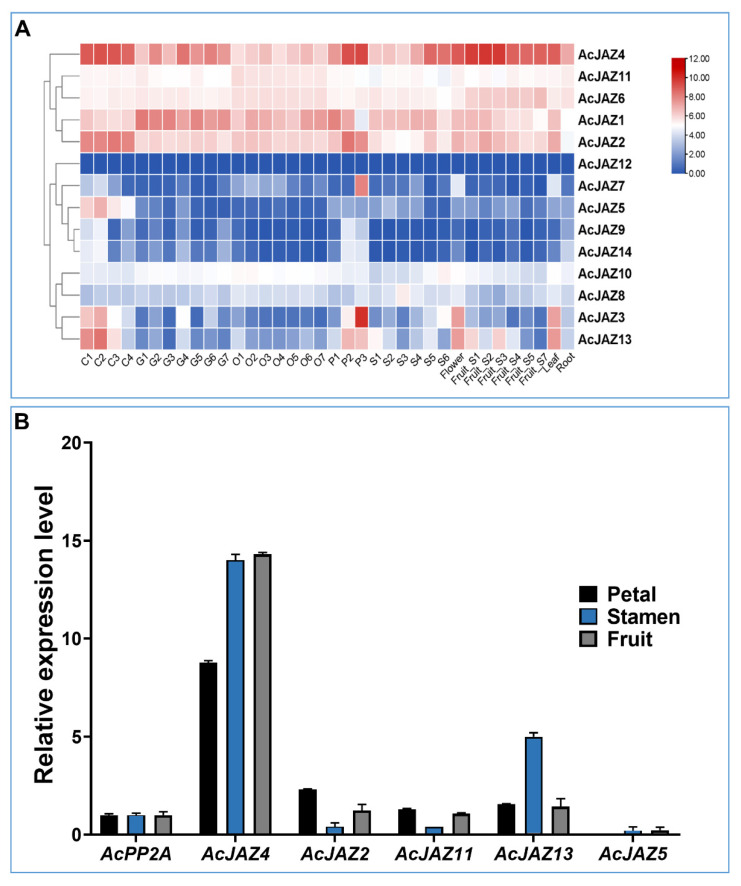
Expression of AcJAZ genes in various tissues. (**A**) Heat-map shows the expression profiles of AcJAZ genes in nine different tissues. C1–C4 (Calyx stage 1–4), G1–G7 (Gynoecium stage 1–7), O1–O7 (Ovule stage 1–7), P1–P3 (Petal stage 1–3), S1–S6 (Stamen stage 1–6), Flower, Fruit _S1–S7, leaf, root. The scale indicates log_10_ transformed gene expression by colors ranging from red (high) to blue (low). (**B**) The expression of AcJAZ4, AcJAZ2, AcJAZ11, AcJAZ13, and AcJAZ5 in petal, stamen, and fruit determined by RT-qPCR. AcPP2A was used as the reference.

FPKM values calculated from RNA-seq generated from nine different tissues were used to create a heatmap and study the expression pattern of *AcJAZ* genes (Figure 3A) [47]. In the samples analyzed, all the *AcJAZ* genes except *AcJAZ12* were expressed. The clustering based on the expression patterns resulted in three groups. The first group consisted of five *AcJAZs* (*AcJAZ1*, *AcJAZ2*, *AcJAZ4*, *AcJAZ6*, and *AcJAZ11*) with relatively high expression levels. The second group had *AcJAZ12*, *AcJAZ7*, *AcJAZ5*, *AcJAZ9*, and *AcJAZ14*, with infrequent representation in the investigated tissues. The third group had four genes (*AcJAZ3*, *AcJAZ8*, *AcJAZ10*, and *AcJAZ13*) with moderate and varied expression levels. *AcJAZ4* had the highest expression level among *AcJAZ* genes, and its expression level was higher in the calyx, petal, and fruit developmental stages 1–3, whereas *AcJAZ12* was rarely expressed. *AcJAZ7* showed in petal stage 3 (P3)-specific expression, and *AcJAZ5* had a calyx (C1–C4)-specific expression. The expression patterns of *AcJAZ8*, *AcJAZ9*, *AcJAZ10*, and *AcJAZ14* were constantly low in all tissues. Two genes, *AcJAZ3* and *AcJAZ13*, had a similar expression pattern and were highly expressed in the calyx (C1–C3), petals (P2–P3), flowers, and leaves (Figure 3A). The RNA-seq results were further verified for *AcJAZ2*, *AcJAZ4*, *AcJAZ5*, *AcJAZ11*, and *AcJAZ13* expression using qPCR (Figure 3B). Consistent with RNA-seq, the qPCR results also showed constitutively high expression of *AcJAZ4* and low expression of *AcJAZ5* in all the tissues. The expression patterns of these genes in petals, stamen, and fruit also showed a similar expression pattern as observed in the RNA-seq.

### 3.5. AcJAZs Are Involved in Responses to Phytohormone and Abiotic Stress

**Figure 4:**
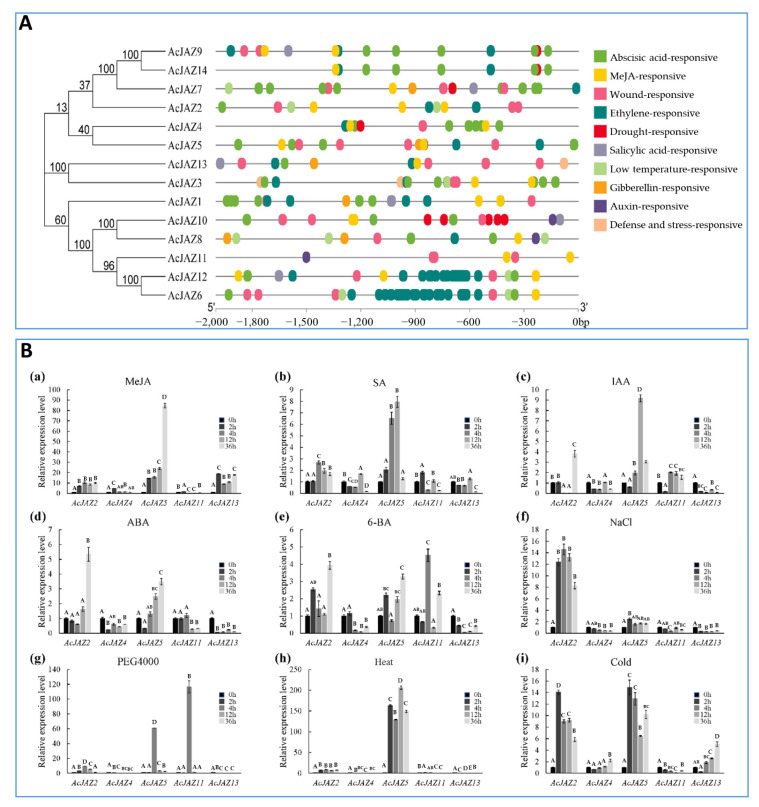
The predicted *cis*-acting elements in the putative promoter regions of *AcJAZs* and the expression of *AcJAZ* genes in response to phytohormone treatments and abiotic stresses. (**A**) The phylogenetic tree of AcJAZs proteins is shown on the left. The abiotic stress-responsive *cis*-acting elements were predicted by the PlantCARE online tool in the 2000 bp sequences upstream of “ATG” of *AcJAZ* genes. The types of *cis*-acting elements were marked by different colors. (**B**) The expression analyses of *AcJAZ* genes under different treatments. One-month-old pineapple seedlings were treated with MeJA (100 μM (**a**)), SA (100 μM (**b**)), IAA (100 μM (**c**)), ABA (100 μM (**d**)), 6-BA (100 μM (**e**)), NaCl (150 mM (**f**)), PEG4000 (15% (**g**)), Heat (45 °C (**h**)), and Cold (4 °C (**i**)). The leaves of the treated seedlings were used for the analysis. *AcPP2A* gene was used for RT-qPCR normalization. Data in (**B**) were analyzed using one-way ANOVA and post hoc Tukey test, and significant differences are indicated by different letters (upper case) at *p* < 0.01.

To predict the abiotic stress-responsive *cis*-elements, the PlantCARE online tool was used to analyze the upstream (2000 bp) sequences of 14 pineapple *AcJAZ* genes and nine putative abiotic stress-responsive *cis*-elements were detected (Figure 4A). Each of the 14 *AcJAZ* genes had between three and eight putative responsive elements except for *AcJAZ11*, and all other *AcJAZs* had one or more ABREs (Figure 4A, Table S4). Not surprisingly, all *AcJAZs* harbor at least one MeJA-responsive element in the 2kb upstream sequences, suggesting their functions in the JA-signaling pathway. The abscisic acid-responsive element (ABRE) and wound-responsive element (WRE3) were the first and third most abundant elements, present in 13 *AcJAZ* genes, suggesting the involvement of AcJAZ in the ABA and wounding response.

We further investigated the expression of *AcJAZ* genes in response to other hormonal and stress conditions. *AcJAZ2*, *AcJAZ4*, *AcJAZ5*, *AcJAZ11*, and *AcJAZ13*, representatives of different groups, were selected for this analysis. The results showed that their expression is regulated by different phytohormones (IAA, ABA, SA, and 6-BA) and abiotic stress treatments (cold, heat, salt, and osmotic stresses) (Figure 4B). *AcJAZ2* and *AcJAZ5* were regulated significantly by SA, IAA, ABA, and 6-BA, and the expression of *AcJAZ11* was induced considerably by 6-BA treatment. Under abiotic treatments, the expression of *AcJAZ2* and *AcJAZ5* was significantly induced by salt, osmotic, heat, and cold stresses. Furthermore, *AcJAZ11* and *AcJAZ13* were markedly induced by osmotic and cold treatments, respectively. Taken together, these results suggest that *AcJAZs* may play important roles in plant responses to phytohormone and abiotic stress, in addition to the JA signaling.

### 3.6. AcJAZs Respond to MeJA in Different Ways

**Figure 5:**
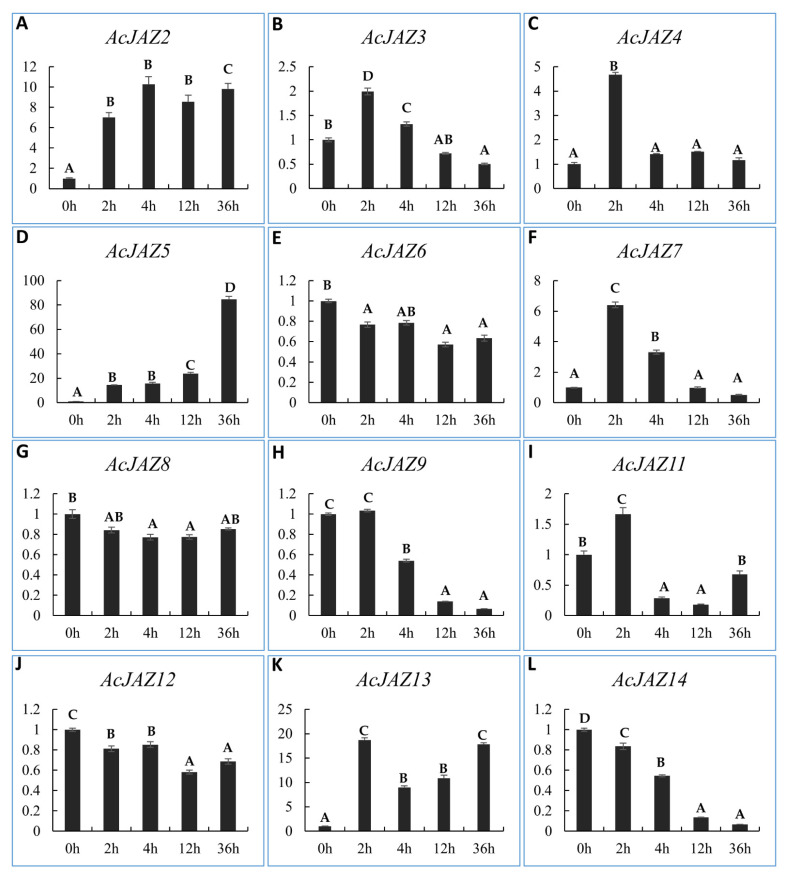
The expression analyses of *AcJAZ* genes under MeJA treatment. The expressions of most *AcJAZ* genes except *AcJAZ1* and *AcJAZ10* were analyzed by RT-qPCR (**A**–**L**). The leaves and roots from one-month-old pineapple seedlings treated with MeJA for 0 h, 2 h, 4 h, 12 h, and 36 h were used to prepare RNA samples. *AcPP2A* gene was used as a reference for RT-qPCR normalization. The upper case letters A, B, C, D indicated the significant difference (*p* < 0.01) among the samples of different time points. Any of the two samples containing the same letter belong to the same group, which would be regarded as not having a significant difference.

JAZ proteins function as repressors of JA-responsive genes in JA signaling [51,52,53,54]. The *cis*-elements analysis showed that all of the *JAZ* genes have MeJA-responsive genes. Therefore, we investigated the expression levels of the 12 *AcJAZs* under MeJA treatment (Figure 5). The results indicated that *AcJAZ6*, *AcJAZ8*, and *AcJAZ12* were insensitive to MeJA treatment and their expression levels did not change compared to control. *AcJAZ3*, *AcJAZ4*, *AcJAZ7*, *AcJAZ11*, and *AcJAZ13* were the early-responsive genes, with a high expression level after 2 h of treatment. *AcJAZ2* and *AcJAZ5* could be considered constant MeJA-responsive genes as their expressions were continuously increased at different time points after treatment. Interestingly, two genes *AcJAZ9* and *AcJAZ14*, were repressed and showed a decreasing expression tendency after the treatment (Figure 5).

### 3.7. Protein–Protein Interaction (PPI) Analysis Revealed the Conserved Interactions among the Central JA-Signaling Regulators in Pineapple

**Figure 6:**
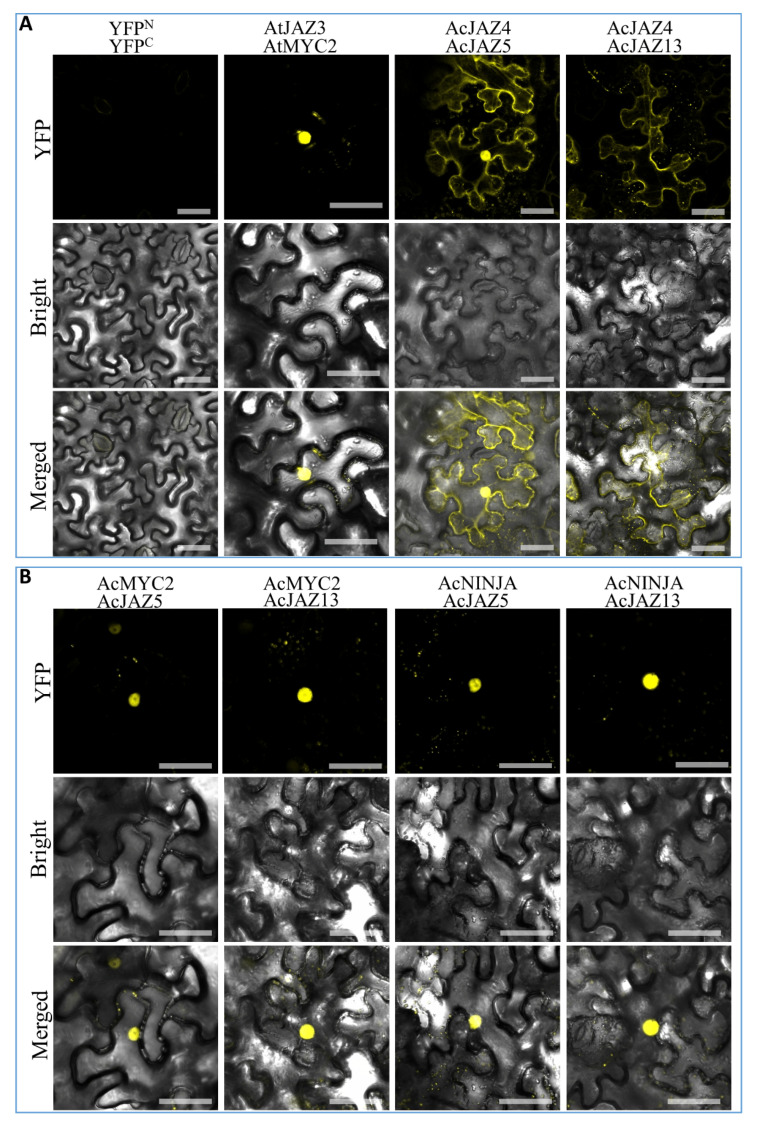
The protein–protein interactions among central regulators of the JA-signaling pathway in pineapple. The images were taken under detecting YFP fluorescence (on the top), bright field (in the middle), and combining fluorescence and bright field (merged images, on the bottom). (**A**) Negative control (the empty vectors of YFP^N^ and YFP^C^ were transiently coexpressed in tobacco leaves), Positive control (construct pairs of YFP^N^-AtJAZ3 and YFP^C^-AtMYC2 were used as a positive control), and BiFC identification of the interactions of YFP^N^-AcJAZ4 with YFP^C^-AcJAZ5 and YFP^C^-AcJAZ13. (**B**) BiFC identification of the interactions of YFP^N^-AcMYC2 with YFP^C^-AcJAZ5 and YFP^C^-AcJAZ13, and YFP^N^-AcNINJA with YFP^C^-AcJAZ5 and YFP^C^-AcJAZ13. Scale bar = 50 μm.

The transcription factor MYC2 plays a crucial role in the JA-signaling pathway, which drives the expression of JA-responsive genes [24,55,56], whereas JAZs bind with MYC2 and inhibit its activity, thereby turning off the expression of JA-responsive genes. We identified the pineapple MYC2 gene (*Aco018875.1*), *AcMYC2* (Table S5), and investigated its interactions with AcJAZ4, AcJAZ5, AcJAZ11, and AcJAZ13 by BiFC. We found that only AcJAZ5 and AcJAZ13 interacted with AcMYC2 in nuclei of tobacco leaf cells (Figure 6 and Figure S4, which confirmed the canonical function of JAZs in pineapple. In *Arabidopsis*, JAM1 is reported as a negative regulator of JA signaling that competitively binds to the promoters of JA-responsive genes and attenuates JA signaling [24,57,58,59,60]. Furthermore, several JAZs could interact with JAM proteins and repress the expression of their targets such as those of MYC2 transcription factors. We verified this regulatory mechanism in pineapple by identifying the *JAM1* (*Aco005839.1*) gene, *AcJAM1* (Table S6), and validating its interactions with AcJAZs using BiFC. AcJAM1 and AcJAZs (AcJAZ5 and AcJAZ13) showed a clear interaction in BiFC experiments, and the signal was detected in nuclei of tobacco leaf cells (Figure S4). Novel Interactor of JAZ (NINJA) is another protein that interacts with JAZs directly and recruits the co-repressor TOPLESS (TPL) [29]. Thus, the pineapple *NINJA* gene (*Aco006735.1*) *AcNINJA* was identified (Table S7), and the interactions between AcNINJA and AcJAZs were also investigated. The results showed that both AcJAZ5 and AcJAZ13 interacted with AcNINJA in nuclei of leaf cells, but no signal was detected from the interaction of AcNINJA with AcJAZ4 or AcJAZ11 (Figure 6 and Figure S4 and Table S8). In addition, homo- and heteromeric interactions between JAZ proteins widely exist in different species, including Arabidopsis, rubber tree, and cotton [61,62,63]. We analyzed and detected the heteromeric interactions of AcJAZ4 with AcJAZ5 and AcJAZ13, but not AcJAZ11 (Figure 6 and Table S8). The signal was localized in the cytoplasm and nuclei (Figure 6). Taken together, the PPI analysis revealed the interactions among the central JA-signaling regulators in pineapple and suggested the conservation of the JA-signaling pathway in plants. 

## 4. Discussion

**Figure 7:**
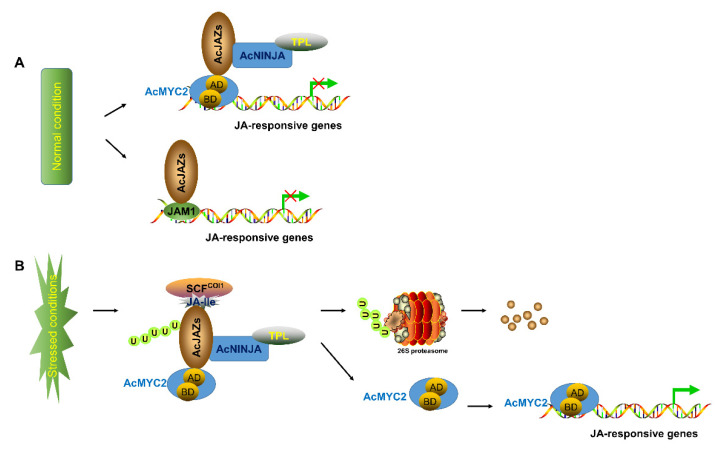
Model of JA-signaling pathway in pineapple. The model indicates the core signal transduction mechanism of jasmonic acid (JA) signaling. (**A**) suggests that AcJAZ repressors bind to AcMYC2 or AcJAM1 to inhibit the expression of JA-responsive genes in pineapple under normal condition. (**B**) shows that the Skp1/Cullin1/F-box protein COI1 (SCF^COI1^) complex mediates the transfer ubiquitin to AcJAZs for ubiquitination and 26S proteasome-mediated degradation, resulting in the release of transcription factors (TFs) such as AcMYC2 and the activation of JA-responsive genes in pineapple.

The JA-signaling pathway is one of the critical pathways involved in plant response to abiotic and biotic stresses. The basic helix–loop–helix transcription factors MYC2, MYC3, MYC4, and MYC5, are key regulators in this pathway which initiate the expression of JA-responsive genes, whereas the JAM1 transcription factor negatively regulates the pathway [24,55,56,57,58,59]. JAZ proteins repress MYC2 and JAM1 by interacting via the C-terminal Jas domain and N-terminal JIDS motif of MYC2 and JAM1. JAZ proteins also interact with NINJA and other JAZ proteins via the TIFY domain [61,62,63]. NINJA, a well-defined transcription co-repressor, interacts with TPL through its EAR domain to recruit the repressive CDK8 Mediator complex and Histone Deacetylase 19 (HD19), thus repressing the expression of target genes [59,64,65,66]. Although the pineapple genome has been deciphered [50], the JA-signaling pathway in pineapple has not been well characterized. In this study, the main regulators involved in JA signaling, including 9 *MYCs*, 8 *NINJAs*, 5 *JAM1s*, and 14 *JAZs*, were identified in pineapple (Tables S5–S7). Based on the gene-expression analysis of the *AcJAZ* gene family (Figure 4 and Figure 5) and protein-interaction results among the main regulators (Figure 6), we propose a hypothetical model of the JA-signaling pathway in pineapple, which is similar to that of Arabidopsis (Figure 7). However, it is worth mentioning that, *AcJAZ2*, *AcJAZ4*, *AcJAZ5*, *AcJAZ11*, and *AcJAZ13* were the early JA-response genes in pineapple, and might play essential roles in pineapple JA signaling.

Based on the F153 genome data [50], we identified 14 gene members in the pineapple *JAZ* gene family (Table 1). A total of 42 JAZ proteins from pineapple and two species, rice (15) and Arabidopsis (13), representing monocot and dicot plants, were used for comparative and phylogenetic analyses [67,68]. The number of JAZ proteins in the pineapple genome was similar to those in rice and Arabidopsis, indicating that the *JAZ* gene family in those three species might encounter similar genome-wide duplication events. According to the Neighbor-Joining (NJ) phylogenetic tree, the 42 JAZ proteins from three species were clustered into six groups (Figure 1B). Group 6 only contained the JAZs from rice, while groups 1, 4, and 5 only had one JAZ from rice, reflecting the evolutionary differences among these three plants. There was a closer relationship between Arabidopsis and pineapple. Furthermore, the members in different pineapple *JAZ* gene family clusters showed significant divergences in exon–intron organization, sequence length, and motifs. Still, those in the same group were very similar for these characteristics (Figure 2A). In addition, the divergence in the motif composition and gene structure provided additional evidence to support phylogenetic groupings.

The JAZ proteins harbor two domains, Jas and TIFY, which interact either with MYC transcription factors when the JA-signaling pathway is not active or with the JA-Ile-SCF^COI^ complex when the pathway is activated. However, the TIFY domain mediates the interactions with NINJA and the formation of homo- and hetero- dimers within the JAZ subfamily [61,62,63]. Pfam results revealed that these AcJAZ proteins possessed a conserved TIFY domain and a Jas domain (Figure 2B). It has been reported that the TIFY domain has several deviations in plants [28,32]. Multiple sequence alignments indicated that the AcJAZ proteins also had several TIFY deviations (Figure S1A). The core sequence of “TIFY” was replaced with “TISF” in AcJAZ6, “TLSF” in AcJAZ11 and AcJAZ12, “TLLF” in AcJAZ8, and “TLLY” in AcJAZ10 (Figure S1A). The structural diversity of the core “TIFY” domain in pineapple JAZ proteins suggests functional diversity among the family members. In contrast, the sequences of the Jas domain among the JAZs in pineapple were more conserved (Figure S1B). Most of the *JAZ* genes are also JA-responsive genes in the JA-signaling pathway. For instance, *AtJAZ10* was expressed immediately after JA treatment [69,70]. The results from MeJA treatment indicate that *AcJAZs* are JA-responsive genes, among which *AcJAZ3*, *AcJAZ4*, *AcJAZ7*, *AcJAZ11*, and *AcJAZ13* are transiently responsive, while *AcJAZ2*, *AcJAZ5*, and *JAZ13* are constantly JA-responsive. It has been reported that *OsJAZ8* could be induced by the JA signal to confer resistance to bacterial blight [71]. Phylogenetic analysis showed that *AcJAZ3*, *AcJAZ13*, and *OsJAZ8* are in the same subcluster, suggesting that MeJA-induced *AcJAZ3* and *AcJAZ13* might also respond to biotic stress. Under MeJA treatment, the expression of *AcJAZ3*, *AcJAZ4*, *AcJAZ7*, *AcJAZ11*, and *AcJAZ1* were transiently up-regulated, and that of *AcJAZ2* and AcJAZ5 constantly up-regulated (Figure 5). The JA-responsive expression of AcJAZ genes in pineapple also provides evidence for the negative feedback loop of the JA-signaling pathway.

The JA-signaling pathway has also been reported to be involved in various abiotic stresses. A previous study showed that *OsJAZ1* is strongly up-regulated under drought stress, and its overexpression of *OsJAZ1* negatively modulates the drought resistance [72]. *Cis*-acting regulatory elements in the promoter regions play a critical role in regulating gene expression by controlling the affinity of transcription factors. In this study, the *cis*-acting elements related to phytohormones (MeJA, SA, IAA, ABA, GA, and ETH) and stress responses (wound, osmotic, and low temperature) were discovered in the putative promoter regions of the pineapple *JAZ* genes (Figure 4). The expression profiles of *AcJAZs* under phytohormone (MeJA, IAA, ABA, SA, and 6-BA) and abiotic (cold, heat, salt, and osmotic) treatments (Figure 4) suggest that *AcJAZ* genes play a pivotal role in plant response to abiotic stresses.

## 5. Conclusions

In this study, 14 pineapple *JAZ* genes were identified and classified into five groups. The AcJAZ genes shared a similar gene structure and motif arrangement. The conservative motifs Jas and TIFY were present in all AcJAZs with their specialized functions. The putative promoter regions of *AcJAZs* contain between three and eight abiotic stress-responsive *cis*-acting elements. The expression patterns of AcJAZs in different tissues and in response to various abiotic stresses suggested that AcJAZs were associated with plant development and differentiation and significant response to different stresses and phytohormones. This finding indicates that AcJAZs may be involved in multiple biological processes during growth and development as well as resisting stresses. Moreover, the BiFC identification of protein interactions of AcJAZs with the central JA-signaling regulators suggested that AcJAZs and other vital players function in the JA-signaling pathway in response to abiotic stresses in pineapple. The results will be helpful in improving breeding for stress tolerance in pineapple.

## Figures and Tables

**Table 1:** Information of Pineapple JAZs, including gene ID, chromosome, locations, isoelectric point (pI), molecular weight (MW) protein length, CDS length, and exon number.

Gene Name	Gene ID	Chr.	Location	Protein Length (aa)	MW (Da)	PI	CDS Length (bp)	Exon Number
*AcJAZ1*	*Aco009689.1*	01	352,698–360,662	395	43,014.82	9.32	1188	10
*AcJAZ2*	*Aco026631.1*	02	6,464,776–6,477,071	238	25,303.57	8.79	714	6
*AcJAZ3*	*Aco018982.1*	02	10,545,290–10,547,516	210	23,796.04	6.98	633	5
*AcJAZ4*	*Aco001047.1*	02	14,641,664–14,650,015	535	56,897.76	9.65	1608	7
*AcJAZ5*	*Aco004646.1*	05	4,735,309–4,740,870	161	16,867.08	8.89	486	5
*AcJAZ6*	*Aco021314.1*	06	2,371,601–2,382,733	322	34,516.57	6.36	969	7
*AcJAZ7*	*Aco005108.1*	07	2,120,529–2,122,794	179	19,892.72	9.55	540	3
*AcJAZ8*	*Aco005280.1*	07	3,669,167–3,673,784	298	32,396.31	5.32	897	8
*AcJAZ9*	*Aco008727.1*	09	1,878,016–1,880,488	174	20,210.45	9.91	525	3
*AcJAZ10*	*Aco000199.1*	12	3,779,203–3,783,099	347	37,278.26	5.58	1044	9
*AcJAZ11*	*Aco006545.1*	14	1,473,910–1,479,052	172	18,381.44	5.58	519	3
*AcJAZ12*	*Aco009467.1*	19	55,884–56,646	195	21,244.75	5.84	588	3
*AcJAZ13*	*Aco023684.1*	20	8,890,114–8,892,147	225	24,753.96	7.86	678	5
*AcJAZ14*	*Aco030609.1*	Scaffold_1707	10,480–12,912	173	20,072.2	9.8	522	3

## Data Availability

All data have been provided in the manuscript as main figures and tables or as Supplementary Materials.

## Supplementary Materials

The following supporting information can be downloaded at: https://www.mdpi.com/2079-7737/11/3/445/s1, Figure S1. The alignment of 14 AcJAZ proteins showed the tify motif and Jas domain; Figure S2. Motif logos of AcJAZs; Figure S3. Motifs in AcJAZ identified by Pfam; Figure S4. The protein–protein interactions among central regulators of the JA-signaling pathway in pineapple (continued). Table S1. The list of RT-PCR Primers; Table S2. Prediction of subcellular localization of AcJAZ proteins; Table S3. Prediction of subcellular localization of OsJAZ proteins. Table S4. The cis-acting abiotic responsive elements in Pineapple JAZ genes; Table S5. MYC2 genes in pineapple; Table S6. JAM1 genes in pineapple; Table S7. NINJA genes in pineapple; Table S8. BiFC identification of protein–protein interactions among central regulators of the JA-signaling pathway in pineapple; Supplemental File S1. All protein sequences used for phylogenetic analysis in this study; Supplemental File S2. The motif information of pineapple JAZ proteins.
