# Correction: Ye et al. Investigation of the JASMONATE ZIM-DOMAIN Gene Family Reveals the Canonical JA-Signaling Pathway in Pineapple. *Biology* 2022, *11*, 445

**DOI:** 10.3390/biology11101385

**Published:** 2022-09-23

**Authors:** Li Ye, Ling Cao, Xuemei Zhao, Xinya Guo, Kangzhuo Ye, Sibo Jiao, Yu Wang, Xiaoxue He, Chunxing Dong, Bin Hu, Fang Deng, Heming Zhao, Ping Zheng, Mohammad Aslam, Yuan Qin, Yan Cheng

**Affiliations:** 1 State Key Laboratory of Ecological Pest Control for Fujian and Taiwan Crops, College of Plant Protection, Fujian Agriculture and Forestry University, Fuzhou 350002, China; 2 Fujian Provincial Key Laboratory of Haixia Applied Plant Systems Biology, Center for Genomics and Biotechnology, College of Life Science, Fujian Agriculture and Forestry University, Fuzhou 350002, China; 3 College of Agriculture, Fujian Agriculture and Forestry University, Fuzhou 350002, China; 4 Guangxi Key Lab of Sugarcane Biology, College of Agriculture, Guangxi University, Nanning 530004, China

The authors would like to make the following correction to the published paper [1].

## 1. Error in Figures

In the original publication, there was a mistake in Figure 1 as published. Figure 2 was reused, and Figure 1 was missed out by mistake during the revision process. The corrected Figure 1 appears below. 

## 2. Text Correction

In the original publication, there was a mistake in the subtitle of 2.8. “Identification of JAZ Gene Family in Pineapple” as published. The typo was introduced during the reordering of the subsections. The correct subtitle should be “*2.8. The Cellular Localization of AcJAZ Proteins*”.

The authors apologize for any inconvenience caused and state that the scientific conclusions are unaffected. This correction was approved by the Academic Editor. The original publication has also been updated.

## Figures and Tables

**Figure 1:**
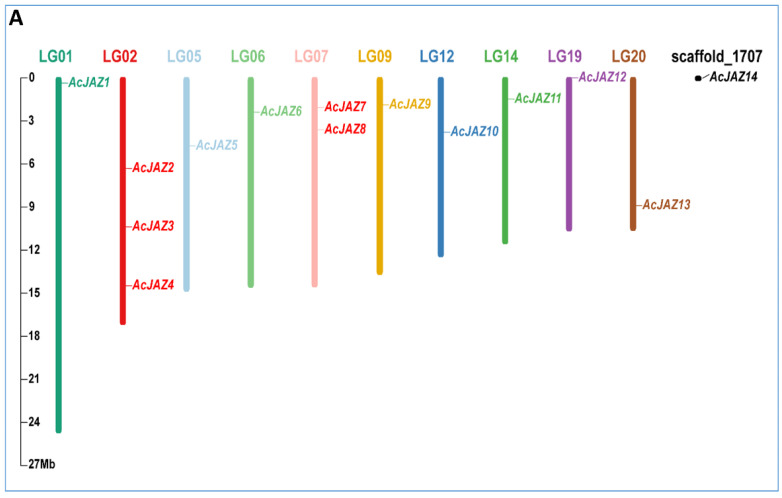

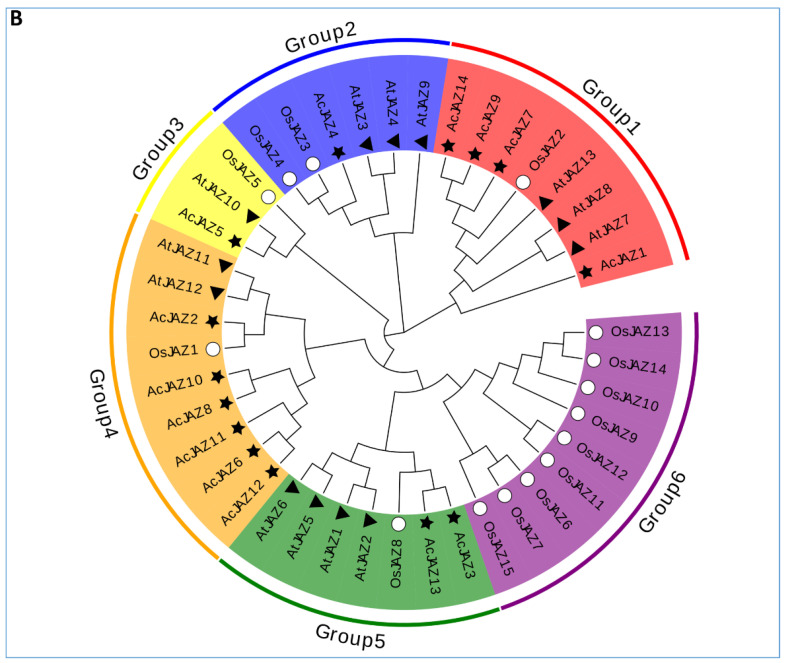
The JAZ gene family in pineapple. (**A**) Chromosomal locations of JAZ genes in pineapple. The respective chromosome number is indicated by different colors at the top of each chromosome. The scale on the left is for chromosomes in megabases (**B**) Phylogenetic relationship of JAZ proteins from *Arabidopsis thaliana*, *Oryza sativa*, and pineapple. The phylogenetic tree was created using the neighbor-joining method with 1000 bootstrap replicates by MEGA 7. The diverse groups of JAZ proteins are marked with different colors. The JAZ proteins of *A. thaliana*, *O. sativa*, and pineapple are represented by black triangles, white circles, and black stars, respectively.

## References

[1] Ye L., Cao L., Zhao X., Guo X., Ye K., Jiao S., Wang Y., He X., Dong C., Hu B. (2022). Investigation of the JASMONATE ZIM-DOMAIN Gene Family Reveals the Canonical JA-Signaling Pathway in Pineapple. Biology.

