# Supplementary material for: Investigation of the JASMONATE ZIM-DOMAIN Gene Family Reveals the Canonical JA-Signaling Pathway in Pineapple
Source: Biology (Basel). 2022 Mar 15;11(3):445. doi: 10.3390/biology11030445 (PMC8945601; doi:10.3390/biology11030445)
Supplement: Supplementary file 1 [file biology-11-00445-s001.zip › Supplemental Tables and Figures.pdf]

**Supplementary Table and Figures**

**Table S1.** The list of RT-PCR Primers.

| Gene | Forward Primer (5′-3′) | Reverse Primer (5′-3′) | Function |
|---|---|---|---|
| *AcJAZ1* | CTTTGGACGTATGCTGCAGA | TCTGCAGCATACGTCCAAAG | RT-qPCR |
| *AcJAZ2* | TCGCAGCTCACAATCTTCTAC | GAGCTTGCAGAGCGAACTAT | RT-qPCR |
| *AcJAZ3* | TTGTAGCCTCTTGAGCCAATAC | CTCATTCGTCCCTTCCTTGTT | RT-qPCR |
| *AcJAZ4* | ACAAGGTGGGTCTCTCTCTAA | CAGCATTCTCTGAGGGTTTCT | RT-qPCR |
| *AcJAZ5* | GCAGCGGAAGCAAGTAATGGAG | CTCGCGATTGGCAGCTCTC | RT-qPCR |
| *AcJAZ6* | GGTTCGGTTCCATCCTCTTC | CGGTCGACCTTCCACTAAAC | RT-qPCR |
| *AcJAZ7* | TCTGCCTCCGACTTGGTAT | GATGGCTCTTGCCTGAATCT | RT-qPCR |
| *AcJAZ8* | CTCGGTCACCCCCGAAAAG | GAGGGCTACCTCCTTGCGAAC | RT-qPCR |
| *AcJAZ9* | GCAGCAGCAACAGCAGCAGC | CATTTCTCGTCTCGCCATCGATA | RT-qPCR |
| *AcJAZ10* | CGATTCGCTCTTCGACTCATC | CACACCCTCCCAACAGTAATAG | RT-qPCR |
| *AcJAZ11* | AGGGCATTGAGAGGTACGA | ACTCTGTGCGGCAAGTTTAT | RT-qPCR |
| *AcJAZ12* | CTCCGTTTCCCCCGAAAAG | CTACCTGAGAGCAACCTCCTTCC | RT-qPCR |
| *AcJAZ13* | GGTAAGGTCGAGGAATTGATGCG | CTTTCCCTCCTTTTCGCGAGG | RT-qPCR |
| *AcJAZ14* | CAGCAGCAGCAGCAGCGGG | CTCCATTTCTCGCCTCGCCATC | RT-qPCR |
| *AcPP2A* | TTGTCATCGCTTCCTCCAAG | GTGTTGTCCACCACAGTATGA | RT-qPCR |
| *AcJAZ4* | cacgggggactctagaATGGAGCGGGATTTTTT GGG | tcccgggagcggtaccTTAGAGAGAGAGA TGGAGAGAGAGAG | BiFC |
| *AcJAZ5* | cacgggggactctagaATGTCGAAGGCCGCCGC C | tcccgggagcggtaccTTAGGTGGTCAACC TTTCCTTGCGCC | BiFC |
| *AcJAZ13* | cacgggggactctaga ATGGAGAGGGAAAAAACCAAC | tcccgggagcggtacc CTACAAACGAAGCTCATGTTC | BiFC |
| *AcMYC2* | cacgggggactctaga ATGATGGAGGCCTTCATG | tcccgggagcggtacc CTACCTGCAAGGAGAGGG | BiFC |
| *AcNINJA* | cacgggggactctaga ATGGAGGACGAAAATGGCCTC | tcccgggagcggtaccGTTTTGGGCCGATG CTGCTG | BiFC |
| *AcJAM1* | cacgggggactctaga ATGGTGATGGGGAGGCTATG | tcccgggagcggtaccCTGCGACTGGGACT GCGAC | BiFC |

**Table S2. Prediction of subcellular localization of AcJAZ proteins**

| AcJAZs | DeepLoc analysis: scores | | | | Prediction | MULocDeep analysis: scores | | | | Prediction |
|---|---|---|---|---|---|---|---|---|---|---|
| | Nucleus | Cytoplasm | Membrane | Peroxisome | | Nucleus | Cytoplasm | Membrane | Peroxisome | |
| AcJAZ1 | 0.5712 | 0.0113 | 0.0385 | 0.2126 | Nucleus | 0.8517 | 0.1329 | 0.0024 | 0.0024 | **Nucleus** |
| AcJAZ2 | 0.9938 | 0.0061 | 0 | 0 | Nucleus | 0.7764 | 0.0575 | 0.0022 | 0.0018 | **Nucleus** |
| AcJAZ3 | 0.6793 | 0.0101 | 0.0779 | 0.1179 | Nucleus | 0.7206 | 0.0926 | 0.0011 | 0.0019 | **Nucleus** |
| AcJAZ4 | 0.8810 | 0.0103 | 0.0929 | 0.0027 | Nucleus | 0.5278 | 0.1691 | 0.0045 | 0.0018 | **Nucleus** |
| AcJAZ5 | 0.9470 | 0.0397 | 0.0102 | 0.0005 | Nucleus | 0.7679 | 0.0023 | 0.001 | 0.0051 | **Nucleus** |
| AcJAZ6 | 0.9894 | 0.0103 | 0.0002 | 0 | Nucleus | 0.8423 | 0.1112 | 0.0019 | 0.0016 | **Nucleus** |
| AcJAZ7 | 0.9908 | 0.009 | 0.0001 | 0 | Nucleus | 0.8001 | 0.1192 | 0.0022 | 0.0021 | **Nucleus** |
| AcJAZ8 | 0.9970 | 0.0427 | 0.0002 | 0 | Nucleus | 0.8963 | 0.0418 | 0.0026 | 0.0019 | **Nucleus** |
| AcJAZ9 | 0.9951 | 0.0022 | 0.002 | 0.0001 | Nucleus | 0.7391 | 0.1236 | 0.0019 | 0.0018 | **Nucleus** |
| AcJAZ10 | 0.9929 | 0.0069 | 0.0001 | 0 | Nucleus | 0.4406 | 0.0091 | 0.0021 | 0.0015 | **Nucleus** |
| AcJAZ11 | 0.9998 | 0.0002 | 0 | 0 | Nucleus | 0.7853 | 0.1372 | 0.0042 | 0.0019 | **Nucleus** |
| AcJAZ12 | 0.9999 | 0.0001 | 0 | 0 | Nucleus | 0.8354 | 0.041 | 0.0424 | 0.0024 | **Nucleus** |
| AcJAZ13 | 0.8363 | 0.0509 | 0.0584 | 0.0059 | Nucleus | 0.5198 | 0.0331 | 0.0025 | 0.0074 | **Nucleus** |
| AcJAZ14 | 0.9921 | 0.0025 | 0.0036 | 0.0001 | Nucleus | 0.9323 | 0.0292 | 0.0043 | 0.0018 | **Nucleus** |

**Table S3. Prediction of subcellular localization of OsJAZ proteins.**

| **OsJAZs** | **DeepLoc analysis: scores** | | | | **Prediction** | **MULocDeep analysis: scores** | | | | **Prediction** |
|---|---|---|---|---|---|---|---|---|---|---|
| | **Nucleus** | **Cytoplasm** | **Membrane** | **Peroxisome** | | **Nucleus** | **Cytoplasm** | **Membrane** | **Mitochondrion** | |
| **OsJAZ1** | **0.9958** | **0.004** | **0.0002** | **0.0001** | **Nucleus** | **0.6839** | **0.2866** | **0.0019** | **0.0037** | **Nucleus** |
| **OsJAZ2** | **0.9917** | **0.008** | **0.0001** | **0.0001** | **Nucleus** | **0.9943** | **0.0395** | **0.0012** | **0.0016** | **Nucleus** |
| **OsJAZ3** | **0.9307** | **0.065** | **0.0019** | **0.0008** | **Nucleus** | **0.477** | **0.2956** | **0.0179** | **0.0012** | **Nucleus** |
| **OsJAZ4** | **0.9754** | **0.023** | **0.0003** | **0.0007** | **Nucleus** | **0.1322** | **0.6674** | **0.0077** | **0.0024** | **Cytoplasm** |
| **OsJAZ5** | **0.7923** | **0.196** | **0.0038** | **0.0022** | **Nucleus** | **0.7517** | **0.1888** | **0.001** | **0.0022** | **Nucleus** |
| **OsJAZ6** | **0.9861** | **0.014** | **0** | **0.0001** | **Nucleus** | **0.793** | **0.0267** | **0.0017** | **0.0065** | **Nucleus** |
| **OsJAZ7** | **0.9360** | **0.029** | **0.0105** | **0.0132** | **Nucleus** | **0.7086** | **0.0904** | **0.0009** | **0.1257** | **Nucleus** |
| **OsJAZ8** | **0.8515** | **0.046** | **0.0312** | **0.0357** | **Nucleus** | **0.4382** | **0.374** | **0.001** | **0.093** | **Nucleus** |
| **OsJAZ9** | **0.9799** | **0.017** | **0.0007** | **0.0019** | **Nucleus** | **0.3738** | **0.3097** | **0.0009** | **0.0108** | **Nucleus** |
| **OsJAZ10** | **0.7663** | **0.174** | **0.0369** | **0.0024** | **Nucleus** | **0.2317** | **0.2242** | **0.0036** | **0.3879** | **Mitochondrion** |
| **OsJAZ11** | **0.7008** | **0.275** | **0.0108** | **0.0011** | **Nucleus** | **0.5993** | **0.2065** | **0.0009** | **0.0037** | **Nucleus** |
| **OsJAZ12** | **0.6676** | **0.1444** | **0.0896** | **0.0215** | **Nucleus** | **0.3619** | **0.1213** | **0.0017** | **0.1828** | **Nucleus** |
| **OsJAZ13** | **0.9643** | **0.024** | **0.0052** | **0.0018** | **Nucleus** | **0.1703** | **0.0196** | **0.0015** | **0.2418** | **Mitochondrion** |
| **OsJAZ14** | **0.7302** | **0.207** | **0.0224** | **0.021** | **Nucleus** | **0.3191** | **0.2839** | **0.0014** | **0.035** | **Nucleus** |
| **OsJAZ15** | **0.9984** | **0.001** | **0.0001** | **0.0001** | **Nucleus** | **0.7406** | **0.0087** | **0.0008** | **0.0163** | **Nucleus** |

**Table S4.** The *cis*-acting abiotic responsive elements in Pineapple *JAZ* genes.

| Gene name | Abiotic responsive elements | | | | | | | | | |
|---|---|---|---|---|---|---|---|---|---|---|
| | ABRE[1] | MeJA[2] | WRE[3] | ERE[4] | DRE[5] | SARE[6] | LTRE[7] | GIRE[8] | AURE[9] | TC-rich[10] |
| *AcJAZ1* | 14 | 4 | 3 | 4 | 0 | 2 | 0 | 1 | 0 | 0 |
| *AcJAZ2* | 2 | 6 | 3 | 3 | 0 | 0 | 2 | 0 | 0 | 0 |
| *AcJAZ3* | 12 | 4 | 2 | 2 | 0 | 0 | 1 | 0 | 0 | 1 |
| *AcJAZ4* | 13 | 4 | 1 | 2 | 1 | 0 | 0 | 0 | 0 | 0 |
| *AcJAZ5* | 12 | 4 | 4 | 2 | 0 | 0 | 0 | 1 | 1 | 0 |
| *AcJAZ6* | 4 | 4 | 4 | 26 | 0 | 0 | 2 | 0 | 0 | 0 |
| *AcJAZ7* | 15 | 2 | 4 | 1 | 1 | 1 | 1 | 1 | 0 | 0 |
| *AcJAZ8* | 5 | 2 | 1 | 1 | 0 | 0 | 4 | 2 | 0 | 0 |
| *AcJAZ9* | 10 | 4 | 2 | 4 | 1 | 1 | 0 | 0 | 0 | 0 |
| *AcJAZ10* | 3 | 4 | 3 | 0 | 5 | 3 | 0 | 0 | 1 | 0 |
| *AcJAZ11* | 0 | 4 | 3 | 0 | 0 | 0 | 0 | 0 | 1 | 0 |
| *AcJAZ12* | 4 | 8 | 2 | 23 | 0 | 1 | 1 | 0 | 0 | 0 |
| *AcJAZ13* | 1 | 2 | 4 | 2 | 0 | 1 | 0 | 1 | 0 | 1 |
| *AcJAZ14* | 9 | 2 | 0 | 3 | 1 | 0 | 0 | 0 | 0 | 0 |

Note: [1] Abscisic acid responsive elements; [2] MeJA-responsive elements; [3] Wound responsive elements; [4] Ethylene responsive elements; [5] Drought responsive elements; [6] Salicylic responsive elements; [7] Low-temperature responsive elements; [8] Gibberellin-responsive elements; [9] Auxin-responsive elements; [10] Defense and stress responsive elements.

**Table S5.** *MYC2* genes in pineapple

| No. | Gene ID | Bit score | E-value |
|---|---|---|---|
| 1 | Aco018875.1 | 498 | 5.00E-169 |
| 2 | Aco018208.1 | 158 | 3.00E-42 |
| 3 | Aco026873.1 | 137 | 8.00E-38 |
| 4 | Aco010845.1 | 135 | 2.00E-33 |
| 5 | Aco005839.1 | 122 | 1.00E-29 |
| 6 | Aco030901.1 | 103 | 3.00E-23 |
| 7 | Aco007275.1 | 102 | 9.00E-23 |
| 8 | Aco012028.1 | 92 | 2.00E-19 |
| 9 | Aco029228.1 | 82 | 4.00E-17 |

**Table S6.** *JAM1* genes in pineapple.

| No. | Gene ID | Bit score | E-value |
|---|---|---|---|
| 1 | Aco005839.1 | 186 | 3.00e-51 |
| 2 | Aco030901.1 | 84.3 | 4.00e-17 |
| 3 | Aco007275.1 | 83.6 | 8.00e-17 |
| 4 | Aco000272.1 | 82.4 | 4.00e-17 |
| 5 | Aco015882.1 | 81.6 | 6.00e-17 |
| 6 | Aco002246.1 | 80.5 | 1.00e-16 |
| 7 | Aco031432.1 | 79.3 | 2.00e-16 |
| 8 | Aco022880.1 | 77 | 8.00e-15 |

**Table S7.** *NINJA* genes in pineapple.

| No. | Gene ID | Bit score | E-value |
|---|---|---|---|
| 1 | Aco006735.1 | 210 | 8e-62 |
| 2 | Aco017364.1 | 72.0 | 5e-14 |
| 3 | Aco012414.1 | 66.2 | 7e-12 |
| 4 | Aco003903.1 | 63.5 | 3e-11 |
| 5 | Aco004854.1 | 60.5 | 4e-10 |

**Table S8. BiFC identification of protein-protein interactions among central regulators of the JA-signaling pathway in pineapple**.

| -YFP$^C$ / -YFP$^N$ | YFP$^C$ | AcJAZ4 | AcJAZ5 | AcJAZ11 | AcJAZ13 |
|---|---|---|---|---|---|
| YFP$^N$ | - | - | - | - | - |
| AcMYC2 | - | - | + | - | + |
| AcNINJA | - | - | + | - | + |
| AcJAZ4 | - | | + | - | + |
| AcJAM1 | - | - | + | - | + |

**Note:** "+" means detected fluorescence signal from the protein-protein interaction by BiFC analysis; "-" represents no fluorescence signal detected from the vector pairs.

**(a)**

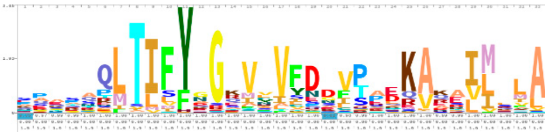

```
AcJAZ1     ..AGQMTIFYDGKINVYDGVMPDKARAIMQLA
AcJAZ2     AAPSQLTIFYNGAVSVYDAIPQEKAQAIMLIA
AcJAZ3     ..NEQLTIFYGGEVLVFDNISGEKYEELIEMA
AcJAZ4     ..LAQLTIFYAGVVNVFDNVPLDKAQELMLFA
AcJAZ5     .ATSPLTIFYNGTVGVFE.LTHDKAKNILRMA
AcJAZ6     ..NNQLTLSFQGEAYVFDSVSPEKVQAVLLLL
AcJAZ7     QQNKQITIFYKGQICVCD.ITEIQARAIITMA
AcJAZ8     AGSNQLTLLFQGEVYVFDSVTPEKVQAVLLLL
AcJAZ9     .ENK.ITIFYDGQICVCD.VTEIQARAIISMA
AcJAZ10    .ASNQLTLLYQGEVYVFESVSPEKVQAVLLLL
AcJAZ11    ...NQLTLSFQGEVYVFDSVTPEK........
AcJAZ12    ..NNQLTLSFQGEAYVFDSVSPEKVQAVLLLL
AcJAZ13    .EKAQLTIFYGGEVLVFDDFSAGKVEELMRMA
AcJAZ14    .ENK.ITIFYDGQICVCD.VTEIQARAIISMA
```

**(b)**

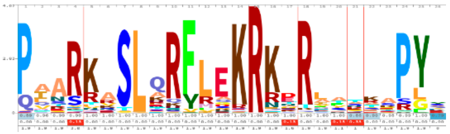

```
AcJAZ1     PTSRKASLQRYLEKRKDR.FK......
AcJAZ2     PIARRHSLQRFLEKRRDRV........
AcJAZ3     PIARRNSLHRFLQKRKHRIN..SKAPY
AcJAZ4     PQARKASLARFLEKRKERMT..NAVPY
AcJAZ5     PIARRKSLQRFLERRKERLT..T....
AcJAZ6     ...RVASLMRFREKRKERNFDKKIRY.
AcJAZ7     ...KR.SLQRFLQKRKARLTAAAAAPY
AcJAZ8     PAKRIASLIRFREKRKERNFDKKIRY.
AcJAZ9     ...KR.SLQRFLQKRKTRIN..AISPY
AcJAZ10    .TSRIASLNRFREKRKERCFDKKIRY.
AcJAZ11    ...RVASLMRFREKRKERNFEKKIRY.
AcJAZ12    ...RVASLMRFREKRKERNFDKKIRY.
AcJAZ13    PLVRRNSLHRFLAKRRERIN..TKAPY
AcJAZ14    ...KR.SLQRFLQKRKTRIN..AISPY
```

**Figure S1.** The alignment of 14 AcJAZ proteins showed the tify motif and Jas domain. (a) tify motif. (b) Jas domain.

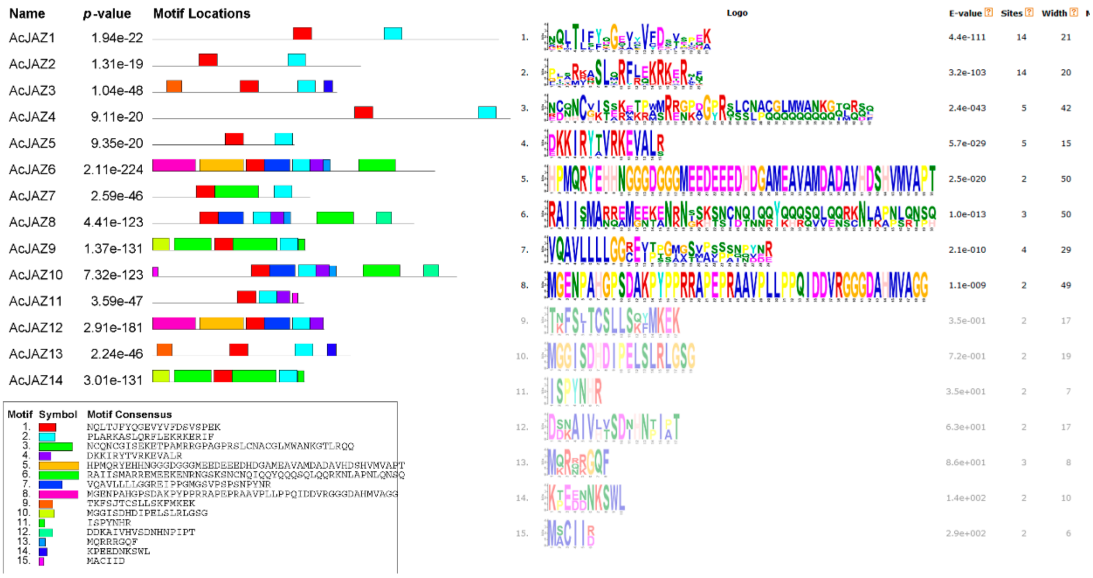


**Figure S2.** Motif logos of AcJAZs. The height of letters within the logos represents the relative frequency of the specific amino acids.

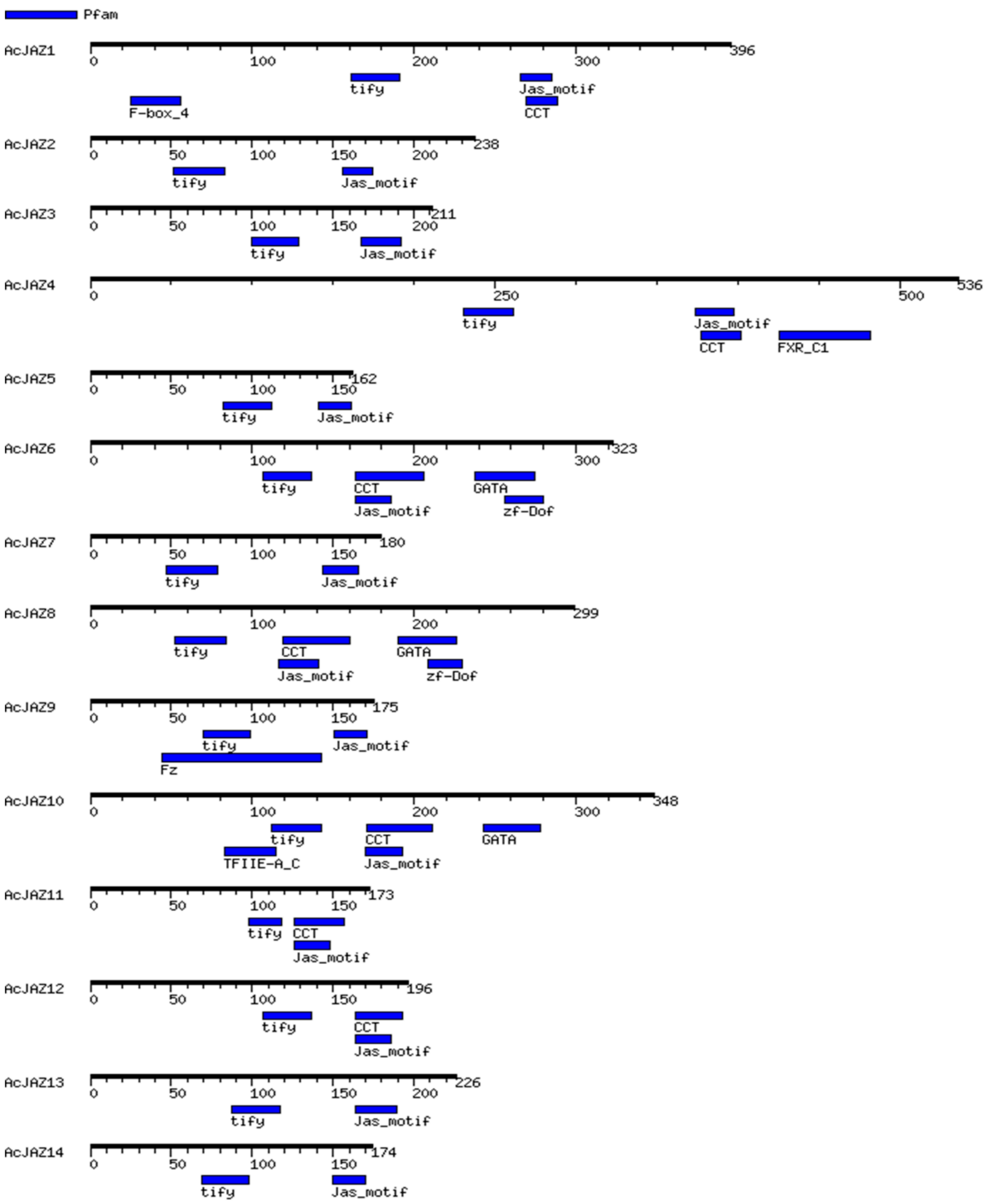


**Figure S3.** Motifs in AcJAZ identified by Pfam.

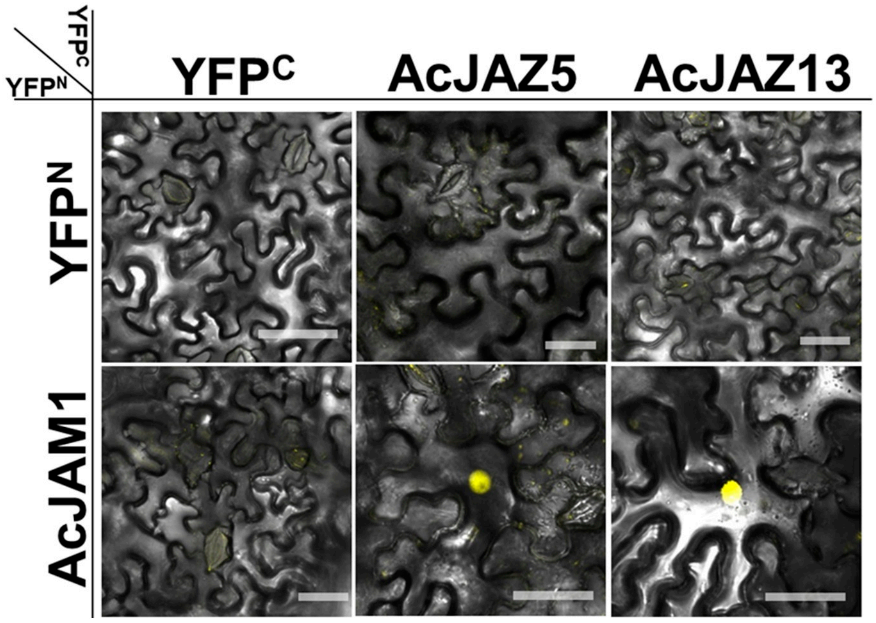


**Figure S4.** The protein-protein interactions among central regulators of the JA-signaling pathway in pineapple (continued). Bar=50μm.
